# How to Contribute to the Progress of Neuroendocrinology: Discovery of GnIH and Progress of GnIH Research

**DOI:** 10.3389/fendo.2018.00662

**Published:** 2018-11-12

**Authors:** Kazuyoshi Tsutsui, Takayoshi Ubuka

**Affiliations:** Laboratory of Integrative Brain Sciences, Department of Biology and Center for Medical Life Science, Waseda University, Tokyo, Japan

**Keywords:** gonadotropin-inhibitory hormone (GnIH), gonadotropin-releasing hormone (GnRH), gonadotropins, melatonin, glucocorticoid, norepinephrine, thyroid hormone, reproduction

## Abstract

It is essential to discover novel neuropeptides that regulate the functions of pituitary, brain and peripheral secretory glands for the progress of neuroendocrinology. Gonadotropin-releasing hormone (GnRH), a hypothalamic neuropeptide stimulating gonadotropin release was isolated and its structure was determined by Schally's and Guillemin's groups at the beginning of the 1970s. It was subsequently shown that GnRH is highly conserved among vertebrates. GnRH was assumed the sole hypothalamic neuropeptide that regulates gonadotropin release in vertebrates based on extensive studies of GnRH over the following three decades. However, in 2000, Tsutsui's group isolated and determined the structure of a novel hypothalamic neuropeptide, which inhibits gonadotropin release, in quail, an avian species, and named it gonadotropin-inhibitory hormone (GnIH). Following studies by Tsutsui's group demonstrated that GnIH is highly conserved among vertebrates, from humans to agnathans, and acts as a key neuropeptide inhibiting reproduction. Intensive research on GnIH demonstrated that GnIH inhibits gonadotropin synthesis and release by acting on gonadotropes and GnRH neurons via GPR147 in birds and mammals. Fish GnIH also regulates gonadotropin release according to its reproductive condition, indicating the conserved role of GnIH in the regulation of the hypothalamic-pituitary-gonadal (HPG) axis in vertebrates. Therefore, we can now say that GnRH is not the only hypothalamic neuropeptide controlling vertebrate reproduction. In addition, recent studies by Tsutsui's group demonstrated that GnIH acts in the brain to regulate behaviors, including reproductive behavior. The 18 years of GnIH research with leading laboratories in the world have significantly advanced our knowledge of the neuroendocrine control mechanism of reproductive physiology and behavior as well as interactions of the HPG, hypothalamic-pituitary-adrenal and hypothalamic-pituitary-thyroid axes. This review describes how GnIH was discovered and GnIH research progressed in this new research era of reproductive neuroendocrinology.

## Introduction

A newresearch field in endocrinology was created by the discovery of neurosecretion, which was named neuroendocrinology. In 1928, Scharrer proposed the concept of neurosecretion that consider hypothalamic neurons terminating in the neurohypophysis produce and secrete neurohormones to regulate endocrine organs. In 1949, Bargmann established this seminal concept proposed by Scharrer. Subsequently, two important hypothalamic neuropeptides, i.e., oxytocin (1) and vasopressin (2), were identified as neurohormones secreted from the neurohypophysis in mammals.

Harris (3) hypothesized that hypothalamic neurons that terminate at the median eminence (ME) may also secrete neurohormones from the ME into the hypophysial portal system to regulate the secretion of anterior pituitary hormones, such as thyroid stimulating hormone (TSH), gonadotropins, i.e., luteinizing hormone (LH) and follicle-stimulating hormone (FSH), growth hormone (GH) and adrenocorticotropic hormone (ACTH). Subsequently, Schally's and Guillemin's groups confirmed this seminal hypothesis by the discovery of important neurohormones, including thyrotropin-releasing hormone (TRH) (4, 5), gonadotropin-releasing hormone (GnRH) (6, 7) and growth hormone-inhibiting hormone (somatostatin) (8), in the brain of mammals. Thus, Schally and Guillemin contributed significantly to the advancement of neuroendocrinology by the discoveries of these neurohormones and they were awarded a Nobel Prize in 1977.

As mentioned above, Schally's (7) and Guillemin's (6) groups discovered a hypothalamic neuropeptide stimulating the release of gonadotropins, i.e., LH and FSH, from the anterior pituitary gland of mammals in the beginning of the 1970s, and the peptide was named GnRH. Subsequent studies demonstrated that GnRH is highly conserved among vertebrates (9–12). Based on extensive studies on GnRH over the next three decades after its discovery, we thought that GnRH is the sole hypothalamic neuropeptide controlling gonadotropin release in vertebrates.

However, in 2000, Tsutsui's group isolated and identified the chemical structure of a novel hypothalamic neuropeptide in quail, an avian species, which inhibits gonadotropin release, and named it gonadotropin-inhibitory hormone (GnIH) (13). The discovery of GnIH opened a new research era of reproductive neuroendocrinology from a novel standpoint. Subsequent studies conducted by Tsutsui's group demonstrated that GnIH is highly conserved among vertebrates from agnathans to humans, acting as an important neurohormone that inhibits vertebrate reproduction [for reviews, see (14–27)]. Tsutsui's group's recent studies have further shown important functions of GnIH beyond the control of reproduction (28, 29). It now appears that GnIH acts in the brain to regulate behavior, including reproductive behavior by regulating the biosynthesis of neurosteroids, such as neuroestrogen, in the brain (29). Therefore, the following 18 years of GnIH research in collaboration with world's leading laboratories has advanced our understanding of the neuroendocrine control mechanism of reproductive physiology and behavior [for reviews, see 14–18, 20–24, 26, 31)].

The discovery of GnIH has changed our understanding about reproductive neuroendocrinology in the past 18 years. Herein this review describes the discovery of GnIH and the progress of intensive research on GnIH focusing on its structure, biosynthesis, biological action, mode of action, and its functional significance in this new research era of reproductive neuroendocrinology. This review also provides a broad overview of the unity and diversity of GnIH structure and biological action and molecular evolution of GnIH in vertebrates.

## Discovery of gnih as a key regulator of reproduction

### History of the discovery of GniH

Tsutsui and colleagues discovered GnIH in the quail brain, while searching a novel hypothalamic neuropeptide that has a C-terminal Arg-Phe-NH_2_ motif (RFamide peptide) (13). Price and Greenberg (32) first identified an RFamide peptide that has a cardioexcitatory effect from the ganglia of the venus clam in the late 1970s. The structure of the isolated peptide was Phe-Met-Arg-Phe-NH_2_ (FMRFamide). Various RFamide peptides that act as neurotransmitters, neuromodulators and hormones had been isolated in other invertebrates after this initial discovery. It was found that FMRFamide-immunoreactive (-ir) neurons were terminating near the anterior pituitary gland in vertebrates (33, 34). Therefore, the existence of unknown hypothalamic RFamide peptides regulating the secretion of anterior pituitary hormones was suggested. This is why Tsutsui's group investigated the existence of an RFamide peptide in the quail brain.

**Figure 1:**
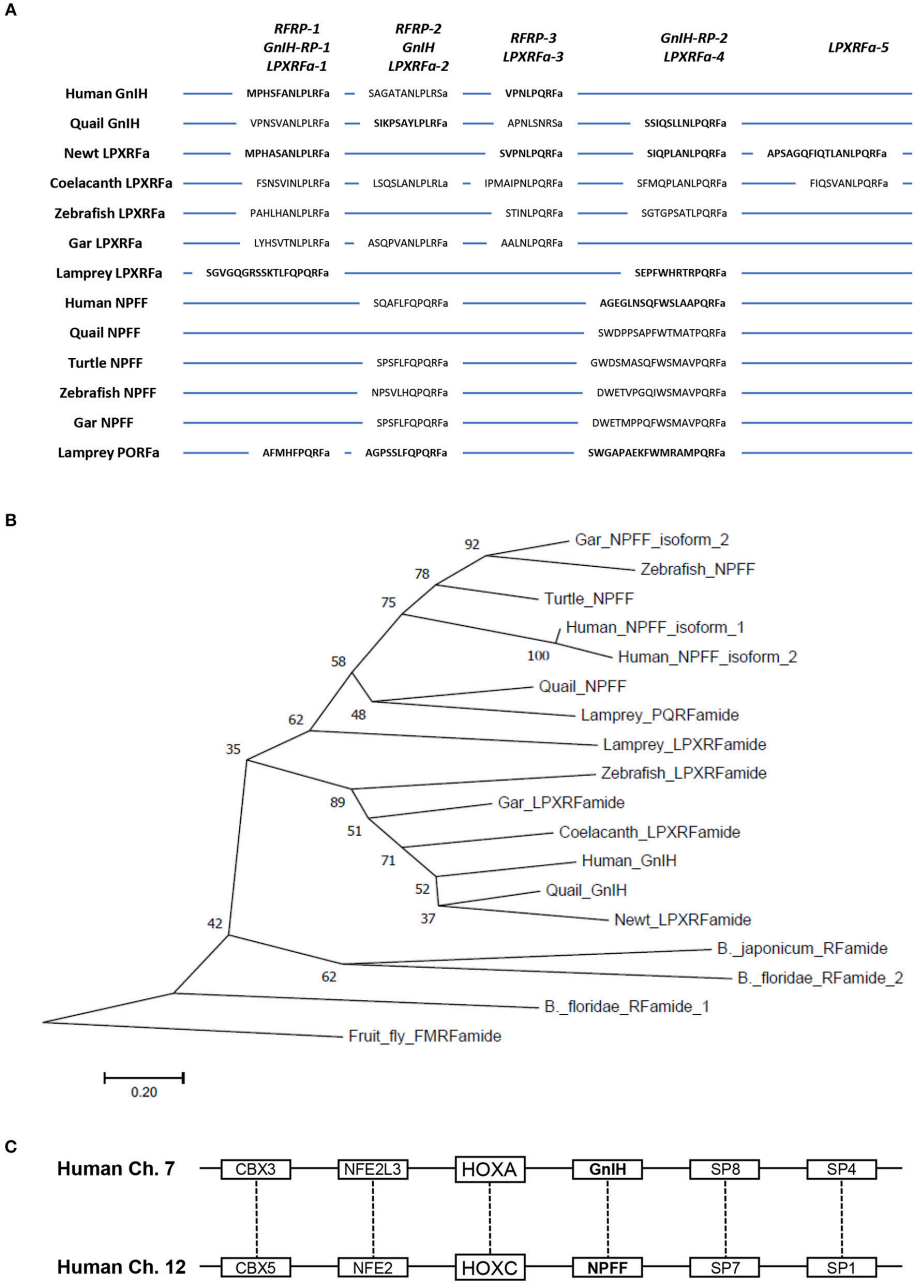
Multiple sequence alignment and phylogenetic analysis of chordate GnIH (LPXRFa) and NPFF (PQRFa) precursor proteins as well as synteny analysis of human GnIH and NPFF genes. **(A)** Multiple sequence alignment of vertebrate GnIH (LPXRFa) and NPFF (PQRFa) precursor proteins highlighting the sequences of identified and predicted biologically active peptides. Precursor protein sequences were aligned by EMBL-EBI Clustal Omega Multiple Sequence Alignment software. Biochemically identified mature peptide sequences are shown in bold. Adapted from Ubuka and Tsutsui (35). **(B)** Phylogenetic tree of chordate GnIH (LPXRFa) and NPFF (PQRFa) precursor proteins. The evolutionary history was inferred using the Neighbor-Joining method. The percentage of replicate trees in which the associated taxa clustered together in the bootstrap test (1,000 replicates) are shown next to the branches. The tree is drawn to scale, with branch lengths in the same units as those of the evolutionary distances used to infer the phylogenetic tree. The evolutionary distances were computed using the Poisson correction method and are in the units of the number of amino acid substitutions per site. Evolutionary analyses were conducted in MEGA7 (36). Accession numbers are human (*Homo sapiens*) GnIH precursor (Human GnIH; NP_071433.3), Japanese quail (*Coturnix japonica*) GnIH precursor (Quail GnIH; XP_015709159.1), Japanese fire belly newt (*Cynops pyrrhogaster*) GnIH precursor (Newt LPXRFamide peptide; BAJ78290.1), West Indian Ocean coelacanth (*Latimeria chalumnae*) GnIH precursor (Coelacanth LPXRFamide peptide; XP_005993154.1), zebrafish (*Danio rerio*) GnIH precursor (Zebrafish LPXRFamide peptide, NP_001076418.1), spotted gar (*Lepisosteus oculatus*) GnIH precursor (Gar LPXRFamide peptide; XP_015213317.1), sea lamprey (*Petromyzon marinus*) GnIH precursor (Petromyzon marinus LPXRFamide peptide; BAL52329.1), Japanese amphioxus (*Branchiostoma japonicum*) GnIH precursor (Branchiostoma japonicum RFamide peptide; BAO77760.1), human NPFF precursor isoform 1 (Human NPFF isoform 1; NP_003708.1), human NPFF precursor isoform 2 (Human NPFF isoform 2; NP_001307225.1), Japanese quail NPFF precursor (Quail NPFF; XP_015705838.1), Western painted turtle (*Chrysemys picta bellii*) NPFF precursor (Turtle NPFF; XP_005307776.1), zebrafish NPFF precursor (Zebrafish NPFF; BAF34891.1), spotted gar NPFF precursor isoform X2 (Gar NPFF isoform 2; XP_015199730.1), sea lamprey NPFF precursor (Petromyzon marinus PQRFamide peptide; BAE79779.1), Florida lancelet (*Branchiostoma floridae*) RFamide precursor 1 (Branchiostoma floridae RFamide peptide 1; XP_002599251.1), Florida lancelet RFamide precursor 2 (Branchiostoma floridae RFamide peptide 2; XP_002609543.1). Fruit fly (*Drosophila melanogaster*) FMRFamide precursor (Fruit fly FMRFamide; NP_523669.2) served as outgroup (root) of the evolutionary tree. **(C)** Synteny analysis of human GnIH and NPFF genes. Paralogous genes are linked by dotted lines. Adapted from Osugi et al. (37).

**Figure 2:**
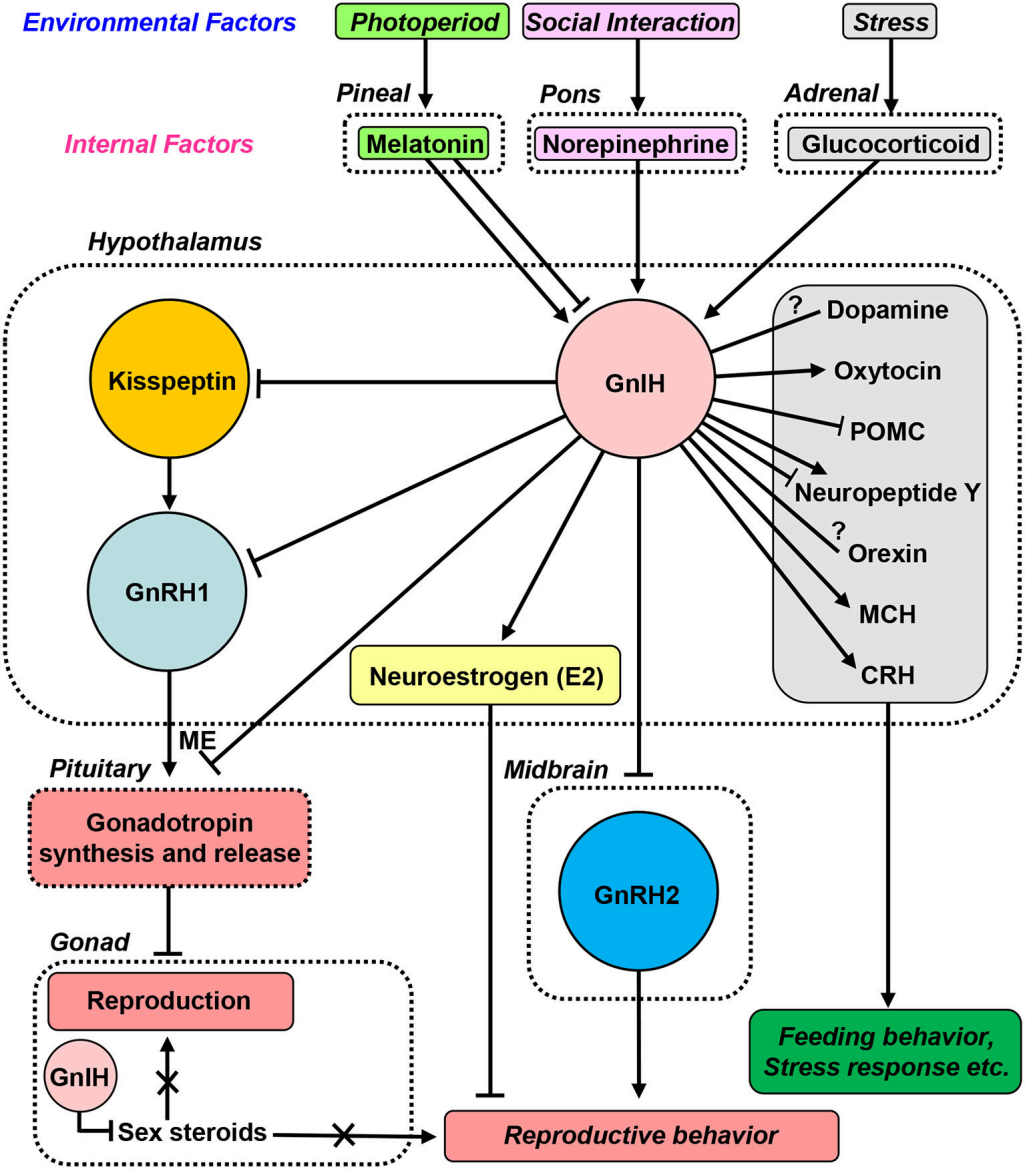
GnIH actions and regulation of GnIH biosynthesis by environmental and internal factors. Cell bodies for GnIH neurons are located in the hypothalamus, paraventricular nucleus in birds and the dorsomedial hypothalamic area in mammals. GnIH neuronal terminals are located to the median eminence (ME) and GnRH1 neurons in the preoptic area in birds and mammals. GnIH receptor is expressed in gonadotropes in the pituitary and GnRH1 neurons in birds and mammals. Thus, GnIH inhibits gonadotropin synthesis and release by directly acting on gonadotropes in the pituitary and by inhibiting the activity of GnRH1 neurons *via* GnIH receptor in birds and mammals. GnIH neurons project not only to GnRH1 neurons but also to kisspeptin neurons in the hypothalamus in mammals. Kisspeptin neurons express GnIH receptor. GnIH and GnIH receptor are expressed in steroidogenic cells and germ cells in gonads, and GnIH acts in an autocrine/paracrine manner to suppress sex steroid production and germ cell differentiation and maturation in birds and mammals. GnIH participates not only in neuroendocrine functions but also in the control of behavior in birds and mammals. GnIH inhibits reproductive behaviors, such as sexual and aggressive behaviors, by acting within the brain. Furthermore, GnIH inhibits reproductive behaviors by stimulating the biosynthesis of neuroestrogen (E2) in the POA. GnIH neurons further project to many other neurons in the brain suggesting multiple actions of GnIH. Environmental factors, such as photoperiod, stress and social interaction, and internal factors, such as melatonin, glucocorticoid and norepinephrine, are important for the control of reproduction and reproductive behaviors. GnIH expression and release are modulated *via* a melatonin-dependent process. Melatonin increases GnIH expression in quail and rats, but melatonin decreases GnIH expression in hamsters and sheep. Stress increases GnIH expression by the actions of glucocorticoids in birds and mammals. Thus, GnIH is a mediator of stress-induced reproductive disruption. The social environment also changes GnIH expression and release mediated by the action of norepinephrine. Stimulatory regulations are shown by arrows, whereas inhibitory regulations are shown by blunt end lines. Lines with a question mark indicate morphological evidence without demonstration of physiological actions. ME, median eminence; POMC, proopiomelanocortin; MCH, melanin-concentrating hormone; CRH, corticotropin-releasing hormone.

In 2000, Tsutsui and colleagues successfully isolated a novel neuropeptide having a C-terminal RFamide motif, Ser-Ile-Lys-Pro-Ser-Ala-Tyr-Leu-Pro-Leu-Arg-Phe-NH_2_ (SIKPSAYLPLRFamide) from the extract of quail brains, using an antibody against RFamide for a competitive enzyme-linked immunosorbent assay on high-performance liquid chromatography (HPLC) fractions (13) (Figure 1A). Importantly, this isolated new RFamide peptide inhibited gonadotropin release from the anterior pituitary of quail actively *in vitro*, which was the first demonstration of a hypothalamic neuropeptide inhibiting gonadotropin release in any vertebrate (13). Given its action on gonadotropin release and its localization in the hypothalamic-hypophysial system, this novel neuropeptide was named GnIH (13) (Figure 2). Cell bodies and terminals for GnIH neurons are located in the paraventricular nucleus (PVN) and ME, respectively, in birds (13). The C-terminal structure of quail GnIH is identical to the chicken LPLRFamide peptide that was reported to be the first RFamide peptide isolated in vertebrates (38), although the chicken LPLRFamide peptide can be a fragment of the chicken GnIH peptide which was found to have a sequence of SIRPSAYLPLRFamide in a recent study (39).

After the discovery of GnIH peptide, GnIH precursor cDNA was sequenced in quail (40) and in other avian species, chicken, sparrows, zebra finches and starlings [for reviews, see (15, 17, 18, 21–23)]. The GnIH precursor cDNA encodes GnIH and GnIH-related peptides GnIH-RP-1 and GnIH-RP-2, which possess a common LPXRFamide (X = L or Q) motif in the C-terminal in all avian species investigated (Figure 1A). GnIH was also isolated as an endogenous mature peptide in starlings (41) and zebra finches (42) and mature GnIH-RP-2 was also isolated in quail (40) (Figure 1A).

GnIH is considered to be a key neuropeptide that inhibits avian reproduction as GnIH inhibits gonadotropin release in avian species [for reviews, see (15, 17, 18, 21–23)]. To investigate the biological action of GnIH *in vivo*, mature male quail were treated with GnIH chronically (43). Chronic treatment with GnIH decreases the expressions of the common α, LHβ and FSHβ subunit mRNAs and the circulating LH concentration. In addition, chronic GnIH treatment induces apoptosis of testicular cells and decreases seminiferous tubules' size in mature male birds (43). In immature birds, GnIH treatment suppresses growth of the testis (43). These findings indicate that GnIH suppresses the development and maintenance of gonads by decreasing synthesis and release of gonadotropin in birds (Figure 2).

### Structure and biological action of GniH in vertebrates

To determine if findings in avian species extend to mammals including primates, Tsutsui and colleagues identified GnIH in the mammalian and primate hypothalamus (44–48). All the identified and predicted mammalian and primate GnIHs possess a common C-terminal LPXRFamide (X = L or Q) motif, like avian GnIH and GnIH-RPs [for reviews, see (15–18, 21–23)] (Figure 1A). Therefore, GnIHs identified in birds, mammals and primates were designated as LPXRFamide peptides based on their structures. On the other hand, mammalian and primate GnIHs are also called RFamide-related peptide 1 and 3 (RFRP-1 and−3) (Figure 1A). Multiple sequence alignment of vertebrate LPXRFamide precursor proteins shows that human RFRP-1 aligns with quail GnIH-RP-1. Human RFRP-3 aligns with a GnIH-like peptide (APNLSNRSa) of quail. On the other hand, quail GnIH aligns with an RFRP-like peptide (SAGATANLPLRSa) of human (Figure 1A). Importantly, it was clearly shown that *in vivo* administration of avian GnIH to female Syrian hamsters centrally or peripherally inhibits LH release (44). Administration of hamster GnIHs (RFRP-1 and−3) centrally inhibits LH release in Siberian hamsters (45). It was also shown that central administration of rat GnIH (RFRP-3) to male rats inhibits LH release (49) and GnRH-elicited gonadotropin release (50, 51). In sheep (52, 53) and cows (54), mammalian GnIH (RFRP-3) administration reduces LH pulse amplitude and inhibits GnRH-elicited gonadotropin synthesis and release. The structure of human GnIH (RFRP-3) was found to be the same as that of ovine GnIH (RFRP-3) (47). Therefore, the biological action of human GnIH (RFRP-3) was examined in the ovine pituitary with Clarke's and Bentley's groups. Importantly, human/ovine GnIH (RFRP-3) clearly inhibits the release of both LH and FSH stimulated by GnRH (52). Thus, it was established that mammalian and primate GnIHs inhibit the synthesis and release of gonadotropin and GnRH-elicited gonadotropin release [for reviews, see (14–18, 21–23)] (Figure 2).

Tsutsui and colleagues identified GnIHs further in reptiles, amphibians and fish to place GnIH findings into a broader perspective in vertebrates. The putative and identified GnIHs in these vertebrate species also possess a C-terminal LPXRFamide (X = L or Q) motif as GnIHs of birds, mammals and primate (55–62) (Figure 1A). It thus appears that GnIHs exist in vertebrates from fish to humans [see (15–25, 27, 30)] for reviews). In fish, Sawada et al. (59) reported that GnIH precursor cDNA encodes three GnIHs, gfLPXRFa-1,−2 and−3 in goldfish. Subsequently, several studies in fish found that goldfish GnIHs have both stimulatory and inhibitory effects on gonadotropin synthesis and release, which may depend on reproductive conditions (63–67). Zhang et al. (67) also showed that GnIH of zebrafish, zfLPXRF-3, suppresses plasma LH levels in goldfish.

Most of the studies in mammals, birds, and fish showed the inhibitory effect of GnIH in the HPG axis. However, several studies in mammals and many studies in fish have shown its stimulatory effect (68). GnIH inhibits LH release in the breeding season when their endogenous LH level is high but stimulates LH release in non-breeding season when their LH level is basal in Siberian hamsters (45). Inhibitory or stimulatory effects of GnIH on the HPG axis depends on the reproductive stages in fish (69). In experiments using higher concentration or longer duration of GnIH administration can stimulate the HPG axis (68). It is considered that the action of GnIH in the HPG axis is modulated by sex-steroid concentration, the action of neuroestrogen synthesized by the activity of aromatase in the brain, estrogen membrane receptor, heteromerization and internalization of GnIH, GnRH, and estrogen membrane receptors (68). The dual action of GnIH in the HPG axis may have a physiological role for reproductive homeostasis according to developmental and reproductive stages of the animal (68).

Although extensive studies have demonstrated that GnIHs are present in the brain of representative species of gnathostomes, the presence of GnIH had not been identified in agnathans, the most ancient lineage of vertebrates (70). Tsutsui and colleagues therefore searched for agnathan GnIH in collaboration with Sower's and Nozaki's groups (71). Because synteny analysis showed the existence of GnIH gene in sea lamprey, Osugi et al. (71) cloned lamprey GnIH precursor cDNA that encodes three GnIHs. Subsequently, these mature GnIHs were isolated from the brain of sea lamprey by immunoaffinity purification and mass spectrometry (71). The isolated lamprey GnIHs possess a common C-terminal PQRFamide motif (71), unlike GnIHs isolated in gnathostomes (Figure 1A).

GnIH neurons exist in the lamprey hypothalamus (71) with GnIH-ir fibers extending to GnRH3 neurons (71). On the other hand, few lamprey GnIH-ir fibers exist in the neurohypophysis compared with lamprey GnRH3-ir fibers (71). Based on the morphology of hypothalamic GnIH neurons, Osugi et al. (71) then analyzed the biological action of lamprey GnIHs on lamprey GnRHs and gonadotropin β subunit expressions and found that lamprey GnIH increases lamprey GnRH3 and gonadotropin β subunit expressions (71). These findings indicate that GnIH exists in the brain of lamprey, which is the oldest lineage of vertebrates and GnIH stimulates the expression of gonadotropin β in the pituitary by acting on GnRH3 neurons (71). Accordingly, GnIH peptide may have emerged as a stimulatory neuropeptide for the regulation of gonadotropin secretion in agnathans and changed its function into an inhibitory neuropeptide during evolution of vertebrates.

### Molecular evolution of GnIH in vertebrates

As described above, most GnIHs are LPXRFamide (X = L or Q) peptides that belong to the RFamide peptide family [for reviews, see (15–25, 27, 30)]. In vertebrates, four more groups, i.e., the prolactin-releasing peptide (PrRP) group, the pyroglutamylated RFamide peptide (QRFP)/26RFamide group, the kisspeptin group, and the neuropeptide FF (NPFF; PQRFamide peptide) group, have been recognized [for reviews, see (15, 16, 25, 27)]. Because the C-terminal structure of NPFF peptides have a C-terminal PQRP motif in vertebrates, which is similar to that of GnIH peptides, the NPFF peptide gene in agnathans was needed to be clarified. Tsutsui and colleagues therefore identified the cDNAs of NPFF precursor in the brain of lamprey and hagfish (72, 73). Importantly, phylogenetic analysis showed that agnathans possess both GnIH and NPFF genes (Figure 1A). Subsequently, agnathan NPFF peptides were identified in sea lamprey and hagfish. The identified agnathan NPFF peptides and GnIH peptides had the same C-ternimal PQRFamide motif (71–73).

Because agnathans have both GnIH and NPFF genes and their mature peptides have the same C-terminal PQRFamide motif (71–73), it was strongly suggested that the GnIH and NPFF genes were derived from a common ancestral gene in protochordates. To investigate this possibility, Tsutsui and colleagues further identified an amphioxus PQRFamide peptide precursor cDNA that encodes three putative PQRFamide peptides (37). Subsequently, the mature peptides of amphioxus PQRFamide were identified by immunoaffinity chromatography and mass spectrometry (37). The amphioxus PQRFamide peptide precursor was suggested to have occurred before the divergence of GnIH and NPFF groups in vertebrates by phylogenetic analysis (37) (Figure 1B). It was found that the amphioxus PQRFamide peptide gene, GnIH gene and NPFF gene of vertebrates have a conserved synteny region around the loci of the genes (37). Vertebrate GnIH and NPFF genes exist near the HOXA and HOXC clusters (Figure 1C), respectively, whereas the amphioxus PQRFamide peptide gene exists near the HOX cluster, suggesting that GnIH and NPFF genes have separated through whole-genome duplication event (37). Based on these findings, it is considered that the amphioxus PQRFamide peptide gene is close to the ancestor of the GnIH and NPFF genes (37, 74). Accordingly, the GnIH and NPFF genes may have separated by whole-genome duplication from an ancestral gene conserved in the protochordate during vertebrate evolution (Figures 1A–C).

## Progression of gnih research focusing on the molecular mechanism of action of gnih on gonadotropin secretion

### Discovery of the receptor for GniH

Tsutsui and colleagues identified the receptor for GnIH in quail to clarify the mode of GnIH action on gonadotropin secretion. The identified GnIH receptor was the G-protein coupled receptor GPR147 (75), which is also named neuropeptide FF receptor 1 (NPFF1). Yin et al. (75) showed that GnIH and GnIH-RPs bind the membrane fraction of COS-7 cells transfected with GnIH receptor cDNA with high affinities. GnIH can act directly on gonadotropes to reduce gonadotropin release in birds, because GnIH receptor is expressed in gonadotropes in the anterior pituitary [for reviews, see (14–18, 21–23, 31)] (Figure 2). In addition, GnIH neurons project to GnRH1 neurons that also express GnIH receptor (76, 41) (Figure 2). Accordingly, it is considered that GnIH acts not only on gonadotropes but also on GnRH1 neurons to inhibit gonadotropin synthesis and release in avian species [for reviews, see (14–18, 21–23, 31)] (Figure 2).

In mammals, Hinuma et al. (77) identified a specific receptor for mammalian GnIH, which is identical to GPR147 and named it OT7T022. Bonini et al. (78) found two GPCRs for NPFF, NPFF1 (identical to GPR147) and NPFF2 (identical to GPR74). GPR147 and GPR74 are paralogous (79). Binding experiments of GnIH and NPFF for GPR147 and GPR74 showed that the affinity of GnIH is higher for GPR147, whereas the agonistic activity of NPFF is potent for GPR74 (78, 80, 81). These findings indicate that the primary receptor for GnIH is GPR147 (NPFF1, OT7T022).

### Mode of GniH action on target cells

Tsutsui and colleagues investigated the effect of GnIH in a mouse gonadotrope cell line, LβT2 to further clarify the cell signaling cascade in gonadotropes triggered by GnIH. Son et al. (82) found that LβT2 cells express GnIH receptor mRNA by RT-PCR. Son et al. (82) further demonstrated that GnIH inhibits GnRH-induced signaling pathways as follows: mouse GnIHs reduced cAMP production and phosphorylation of extracellular signal-regulated kinase (ERK) induced by GnRH (82). Importantly, mouse GnIHs reduced GnRH-induced LHβ expression and LH release (82). Inhibitors of adenylate cyclase (AC) and protein kinase A (PKA) suppressed the stimulatory effect of GnRH on gonadotropin expression, but inhibitor of protein kinase C (PKC) did not (82). These findings indicate that mouse GnIH reduces GnRH-stimulated gonadotropin secretion by interfering with GnRH actions *via* an AC/cAMP/PKA-dependent ERK pathway (82).

Following the discovery of GnIH and its inhibitory action on the hypothalamic-pituitary-gonadal (HPG) axis, kisspeptin was discovered in mammals. In contrast to GnIH, kisspeptin stimulates GnRH neurons and up-regulates the HPG axis in mammals (83–86). Because GnIH neurons project not only to GnRH1 neurons in the preoptic area (POA) but also to kisspeptin neurons in the hypothalamus, GnIH neurons may regulate the activities of both GnRH1 neurons and kisspeptin neurons [for reviews, see (14–18, 21–23, 87)] (Figure 2). Interestingly, GnIH neurons also project to GnRH2 neurons and many other neurons in the brain, which suggest multiple actions of GnIH [for reviews, see (14–18, 21–23)] (Figure 2).

## Progression of GnIH research focusing on multiple actions of gnih

### Gonadal GnIH action on reproduction

Based on extensive GnIH studies, it now appears that GnIH is a key neurohormone for the regulation of reproduction, which reduces gonadotropin synthesis and release by suppressing pituitary gonadotropes and GnRH1 neurons in vertebrates (Figure 2). In addition to the central actions of GnIH, there are reports showing that gonadal GnIH is directly involved in the regulation of gonadal activity locally [for reviews, see (14–18, 21–23, 31)] (Figure 2). Steroidogenic and germ cells in the gonads express both GnIH and GnIH receptor in birds and mammals (88–94). Furthermore, several reports show that GnIH acts in the gonads to suppress sex steroid production and germ cell differentiation and maturation in an autocrine and/or paracrine manner (88–94) (Figure 2).

### Central gnih action on feeding behavior

Importantly, central GnIH participates not only in neuroendocrine control of reproduction but also in behavioral control. Animals use photoperiod to phase breeding with anticipated times of maximal food availability in the environment where energy availability fluctuates (95). When food becomes scarce during the breeding season, reproduction is temporarily inhibited (96, 97). Metabolic challenges such as food deprivation inhibit reproductive axis functioning and sexual motivation (98–102). Tsutsui and colleagues therefore investigated whether GnIH relays metabolic information to the HPG axis and regulates neural feeding circuits [for reviews, see (14, 30)] by the following avian and mammalian studies.

In birds, intracerebroventricular (ICV) injection of GnIH to chicks stimulates food intake (103). ICV injection of anti-GnIH antiserum suppresses fasting induced appetite in support of the stimulatory role of GnIH in feeding (103). Fraley et al. (104) also reported that ICV injection of GnIH decreases plasma LH concentration and increases feeding in adult Pekin ducks. Tachibana et al. (105) further investigated if the orexigenic effect of GnIH involves the opioid and nitric oxide (NO) systems to establish the central mechanism underlying the GnIH action on feeding. The orexigenic effect of ICV injected GnIH is decreased by co-injection of an opioid μ-receptor antagonist β-funaltrexamine, but not by an opioid δ-receptor antagonist ICI-174,864 and an opioid β-receptor antagonist nor-binaltorphimine in chicks (105). In addition, feeding behavior induced by GnIH was not affected by co-injection of a non-selective NO synthase inhibitor (105). McConn et al. (39) also examined the central orexigenic mechanism induced by GnIH in chicks. In the hypothalamus, neuropeptide Y (NPY) mRNA increases, and pro-opiomelanocortin (POMC) mRNA decreases following ICV administration of chicken GnIH (73, Figure 2), McConn et al. (39) further investigated the lateral hypothalamic area (LHA) because ICV injection of GnIH increases c-fos-ir cells in this brain area. They found that melanin-concentrating hormone (MCH) mRNA increases by administration of GnIH (Figure 2). Accordingly, these avian findings indicate that opioid μ-receptor, NPY, POMC and MCH-positive neurons play important roles in the orexigenic response of GnIH (Figure 2).

In mammals, ICV administration of GnIH also increases food intake in rats (49) and sheep (106). Qi et al. (107) showed that GnIH neurons project to NPY, POMC, orexin, and MCH neurons that regulate feeding behavior (Figure 2). Fu and van den Pol (108) showed that chicken and human GnIHs both inhibit POMC neurons and suppress excitation of kisspeptin cells by a mechanism based on opening potassium channels in mouse brain slices (Figure 2). Jacobi et al. (109) found that GnIH inhibits the firing rate in POMC neurons and has an inhibitory effect on action potential activity in NPY neurons in mice (Figure 2). In addition, Jacobi et al. (109) found that NPY neurons have close contacts from GnIH fibers (Figure 2). Thus, these mammalian findings indicate that GnIH participates not only in the regulation of reproduction but also in the regulation of feeding behavior in mammals as in birds.

### Central GniH action on reproductive behaviors

It now appears that central GnIH regulates reproductive behaviors, such as sexual and aggressive behaviors by acting in the brain (29, 110, 111) (Figure 2). In birds, Bentley et al. (110) found that central administration of GnIH inhibits copulation solicitation of female white-crowned sparrows stimulated by the song of males. GnRH2 stimulates copulation solicitation in female white-crowned sparrows stimulated by the song of males (112). As GnIH neurons extend to GnRH2 neurons and GnIH receptor is expressed in GnRH2 neurons in songbirds (41), GnIH may suppress copulation solicitation by suppressing the activity of GnRH2 neurons in songbirds (110) (Figure 2). Ubuka et al. (111) therefore investigated this hypothesis by examining the behavior of male and female white-crowned sparrows modified by RNA interference (RNAi) to the GnIH gene with Wingfield' group. It was found that GnIH RNAi reduces resting time and spontaneous production of complex vocalizations, but stimulates agonistic vocalizations. Furthermore, GnIH RNAi increases song production in male birds challenged by novel male song playbacks (111). These findings indicate that GnIH RNAi induces arousal. Ubuka et al. (111) further found that GnIH mRNA expression in the PVN is negatively correlated with the activity of male and female birds. Importantly, GnIH RNAi decreases the density of GnIH neuronal fibers in the ventral tegmental area in female birds, and the number of GnRH1 and GnRH2 neurons with close GnIH neuronal fiber appositions is negatively correlated with the activity of male birds (111) (Figure 2). Ubuka et al. (29) further demonstrated that GnIH suppresses aggressive behavior in male quail. Accordingly, GnIH may suppress both sexual and aggressive behaviors in birds [see (14, 22, 30, 31) for reviews] (Figure 2).

In mammals, Johnson et al. (49) also found that ICV administration of GnIH inhibits male sexual behavior in rats. Piekarski et al. (113) reported that ICV administration of GnIH reduces sexual motivation and vaginal scent marking but does not suppress lordosis behavior in female hamsters. GnIH administration alters fos expression in the medial POA, medial amygdala and bed nucleus of the stria terminalis, key neural loci implicated in female sexual behavior (113). These mammalian findings indicate that GnIH is an important modulator of female proceptive sexual behavior and motivation (Figure 2). GnIH neurons also project to dopamine neurons in the rhesus macaque brain [(46), Figure 2). Accordingly, GnIH not only acts to regulate the HPG axis but also act to drive the neural circuitry underlying socially-motivated behavior in mammals, as in birds.

### Central GniH action on neurosteroid biosynthesis

There are several reports indicating that neuropeptides and neurosteroids interaction plays an important role in the regulation of brain functions [for a review, see (114)]. Recently, Ubuka et al. (29) found that GnIH increases neuroestrogen synthesis by stimulating the activity of cytochrome P450 aromatase (P450arom) in the quail brain (29) (Figure 2). Importantly, the action of GnIH on the stimulation of neuroestrogen synthesis changes the expression of aggressive behavior in this bird (29) (Figure 2). These results provide a new concept of GnIH that regulates aggressive behavior by modifying the neurosteroid milieu in the brain.

It is established that sexually mature male quail actively fight with intense aggressiveness, unlike female quails (115, 116). Aggressive behavior of male quail is testicular androgen dependent (115–117). However, generally no correlation is observed between aggressiveness and circulating testosterone (T) concentration (117). It is also established that aromatizable androgens, such as T and androstenedione (AD) facilitate aggression in males, but non-aromatizable androgens, such as dihydrotestosterone (DHT) do not, and that T-induced aggression is blocked by administration of P450arom inhibitors (118, 117). Based on these findings, testicular androgen action on aggressive behavior requires aromatization into estrogen (neuroestrogen) in the brain (119, 120, 121).

GnIH neurons project to brain areas, such as the POA (41, 122, 123) and the periaqueductal central gray [PAG; (41)] in birds. GnIH receptor is expressed in the POA (41, 75) and PAG (41). It is known that these brain areas regulate aggressive behavior (124, 125). The POA is also known to be the most critical site of aromatization of testicular androgen by P450arom, and neuroestrogen directly regulates aggressive behavior in male quail (126, 127). Because GnIH decreases aggressive behavior in male birds as described above (29, 111), Ubuka et al. (29) hypothesized that GnIH may decrease aggressive behavior by regulating P450arom activity and neuroestrogen synthesis in the brain (Figure 2). Therefore, Ubuka et al. (29) examined whether GnIH-ir neuronal fibers innervate P450arom cells and P450arom-ir cells express GnIH receptor in the POA in male quail. Ubuka et al. (29) found that abundant GnIH-ir neuronal fibers exist in the vicinity of P450arom-ir cells in the POA and GnIH receptor is expressed in P450arom-ir cells in the POA. They (29) further found that GnIH stimulates the activity of P450arom and increases neuroestrogen concentration in the POA through GnIH receptor (29) (Figure 2). This is the first evidence that GnIH, a hypothalamic neuropeptide, decreases aggressive behavior by stimulating neuroestrogen synthesis in the brain. Subsequently, the effect of centrally administered various doses of estradiol-17β (E2) was tested on the aggressive behavior of male quail. They (29) found that centrally administered higher doses of E2 decreases aggressive behavior (29). This finding indicates that production of neuroestrogen in the brain is essential for the expression of aggressive behavior, but high concentrations of neuroestrogen in the brain decrease aggressive behavior. Accordingly, it is considered that GnIH suppresses aggressive behavior by increasing neuroestrogen synthesis beyond its optimum concentration for the expression of aggressive behavior through activation of P450arom in the brain of male birds (29) (Figure 2).

Ubuka et al. (29) further investigated how GnIH stimulates P450arom activity. P450arom activity is not only controlled by P450arom gene *Cyp19* transcription by steroids in the long term, but also by phosphorylation of P450arom stimulated by neurotransmitters, such as glutamate in the short term (128). Balthazart's group reported that P450arom activity is rapidly down-regulated by phosphorylation in the hypothalamus of male quail (128–132). It is therefore possible that GnIH activates P450arom by dephosphorylation of phosphorylated P450arom. Importantly, Ubuka et al. (29) showed that ICV administration of GnIH decreases phosphorylated P450arom in the POA in the short term, and that the action of GnIH on neuroestrogen synthesis in the POA is abolished by concomitant administration of RF9, a potent antagonist of GnIH receptor (133, 134) or fadrozole, an inhibitor of P450arom (135, 136). These findings indicate that GnIH stimulates neuroestrogen synthesis in the POA by activating P450arom through dephosphorylation after binding GnIH receptor in P450arom cells.

## Progression of GniH research focusing on the regulation of GniH biosynthesis by environmental and internal factors

### Melatonin regulation of GniH biosynthesis under photoperiodic condition

Clarification of the mechanisms regulating GnIH expression in the brain is important for the understanding of the physiological role of GnIH in reproduction. Photoperiodic mammals regulate their reproductive activities depending on the annual cycle of changes in the duration of nocturnal secretion of melatonin (95). There is also evidence that melatonin contributes to the regulation of seasonal changes in gonadotropin secretion and gonadal activity in birds (137–140), despite there is an accepted dogma that reproductive activity of birds is not regulated by seasonal changes in melatonin secretion (141, 142).

Based on this background, Tsutsui and colleagues clarified the action of melatonin on the regulation of GnIH expression in quail, a highly photoperiodic avian species. Ubuka et al. (143) found that removal of melatonin by pinealectomy (Px) and orbital enucleation, minimizes GnIH mRNA and GnIH peptide expressions in the brain of quail (143). By contrast, melatonin administration increases the expressions of GnIH mRNA and GnIH peptide in the quail brain (143). Importantly, a melatonin receptor subtype Mel_1c_ is expressed in GnIH neurons in quail (143), indicating that melatonin induces GnIH expression by acting directly on GnIH neurons (Figure 2). Chowdhury et al. (144) further found that melatonin increases not only GnIH expression but also its release in quail (Figure 2). Interestingly, GnIH release is negatively correlated with plasma LH concentration with diurnal changes (144). In quail, GnIH release is increased in short day (SD) photoperiods, when the duration of nocturnal secretion of melatonin increases (144). Accordingly, it appears that melatonin derived from the pineal gland and eyes acts on GnIH neurons directly *via* Mel_1c_ to induce GnIH expression and release in birds (22, 23, 143, 144) (Figure 2).

In contrast to quail, melatonin decreases GnIH expression in Syrian and Siberian hamsters, both photoperiodic mammals (45, 145, 146) (Figure 2). GnIH expression is reduced in sexually quiescent hamsters exposed to SD photoperiods, compared with sexually active animals under long day (LD) photoperiods. Importantly, these photoperiodic changes in GnIH expression are abolished in Px hamsters and melatonin injections to LD hamsters decrease GnIH expression to SD level (45, 146). Similar seasonal patterns of GnIH expression have been also observed in European (147) and Turkish (148) hamsters, and the semi-desert rodent, Jerboa (149). In addition, there are reports showing that GnIH expression is regulated by melatonin in sheep (150, 151) and rats (152). These results indicate that GnIH expression is regulated photoperiodically by a melatonin-dependent process both in mammals and birds, although species differences exist in the regulation of GnIH expression by melatonin [see (14–18, 22, 23) for reviews] (Figure 2). Furthermore, there is evidence that gonadal GnIH also responds to melatonin directly in a seasonal manner in songbirds (153, 154).

### Glucocorticoid regulation of GniH biosynthesis under stress condition

It is known well that reproduction can be reduced by stress in vertebrates (155). Kirby et al. (156) found that both chronic and acute immobilization stress increases expression of GnIH in the dorsomedial hypothalamic area (DMH) associated with the inhibition of the HPG axis in rats (Figure 2). Adrenalectomy abolishes stress-induced increase in GnIH expression (156). Glucocorticoid receptor (GR) is expressed in GnIH neurons, indicating that adrenal glucocorticoids act directly on GnIH neurons, which may contribute to the increase in GnIH expression (156) (Figure 2). Taken together, these findings imply that GnIH may be an important mediator of stress-induced suppression of reproduction in mammals (156).

Son et al. (157) found that GR is expressed in GnIH neurons in the PVN and corticosterone [CORT; the major glucocorticoid in birds and rodents, (158, 159)] treatment increases GnIH expression, indicating that glucocorticoids can directly regulate GnIH expression in quail (Figure 2).

Furthermore, Son et al. (157) clarified the mechanism of activation of GnIH expression by CORT in rHypoE-23, a GnIH-expressing neuronal cell line, derived from rat hypothalamus. Importantly, GR is expressed in rHypoE-23 cells and CORT treatment increases GnIH expression in these cells (157). It thus appears that stress reduces gonadotropin secretion, at least in part, through the increase in GnIH expression in mammals and birds. Furthermore, there is evidence that gonadal GnIH responds to metabolic challenge as well as stress in a seasonal manner in songbirds (160).

**Figure 3:**
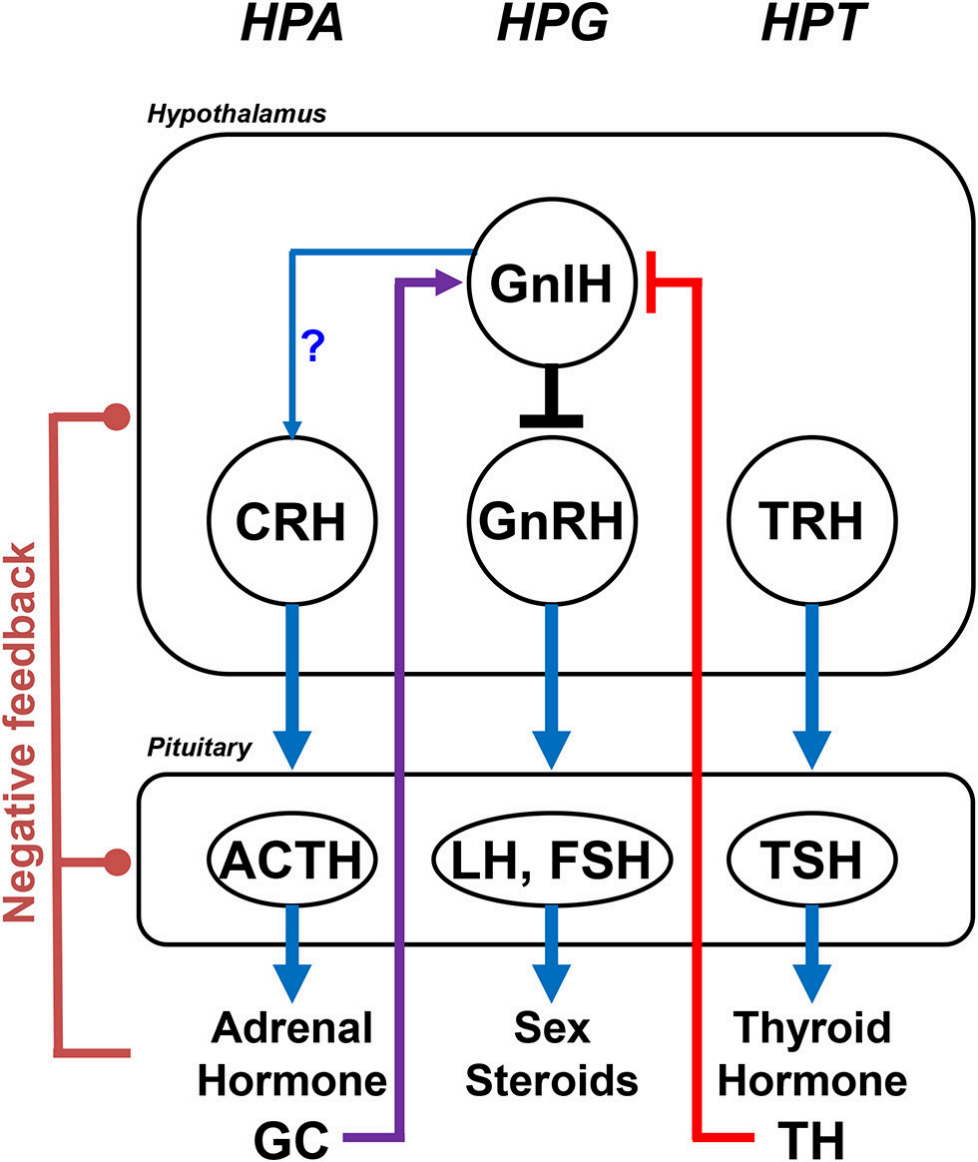
The crosstalk of GnIH with internal factors of different endocrine axes. The interaction between the HPA axis and the axis is mediated by GC and GnIH. Stress suppresses gonadotropin secretion through the increase in GnIH expression in mammals and birds. In addition, the interaction between the axis and the axis was demonstrated. TH-mediated HPG regulation is initiated by inhibiting the expression of GnIH, which acts at the most upstream level of the HPG axis by inhibiting the activity of GnRH neurons to reduce circulating levels of gonadotropins (LH and FSH) and gonadal sex steroids. High concentrations of TH decrease GnIH expression, whereas a lower level of TH increases GnIH expression. The increased GnIH expression induced by hypothyroidism delays pubertal onset. Stimulatory regulations are shown by arrows, whereas inhibitory regulations are shown by blunt end lines. Lines with a question mark indicate morphological evidence without demonstration of physiological actions. HPA, hypothalamic-pituitary-adrenal; HPG, hypothalamic-pituitary-gonadal; HPT, hypothalamic-pituitary-thyroid; GC, glucocorticoid; TH, thyroid hormone.

There are also reports suggesting that GnIH stimulates the hypothalamic-pituitary-adrenal (HPA) axis (107, 161, 162). Qi et al. (107) showed that GnIH neurons project to corticotropin-releasing hormone (CRH) and oxytocin neurons in the PVN in sheep (Figure 2). Kaewwongse et al. (161) showed that ICV injection of RFRPs increases plasma concentration of ACTH and oxytocin in rats. On the other hand, Ullah et al. (162) showed that intravenously injected RFRP-1 increases cortisol level in monkeys. These results suggest that GnIH stimulates the HPA axis, although its mechanism of action is not understood well (Figures 2, 3).

### Norepinephrine regulation of GniH biosynthesis under social environment

In addition to photoperiod and stress, social environment may influence GnIH expression (Figure 2). Calisi et al. (163) manipulated nesting opportunities for pairs of European starlings and GnRH, GnIH, and GnIH mRNA contents in the brain were examined. Fewer numbers of GnIH cells were observed in birds with nest boxes than those without nest boxes, but GnRH did not vary with or without nest box. These findings suggest that GnIH is involved in reproductive function in response to social environment (163).

There are reports showing that plasma T concentrations in male quail is rapidly decreased by the presence of a female bird (164, 165). Based on these observations, Tsutsui and colleagues looked into the neurochemical mechanism of social interaction that alters reproductive physiology (Figure 2). Tobari et al. (28) first found that norepinephrine (NE) release rapidly increases in the PVN of male quail when viewing a conspecific female (Figure 2). GnIH expression increases in the PVN of male quail as well, with associated decreases in plasma LH concentrations, when males view a female (28) (Figure 2). Subsequently, Tobari et al. (28) showed that NE application to male quail increases GnIH release. GnIH neurons express α2A-adrenergic receptor and GnIH neurons are innervated by noradrenergic fibers (28). These findings indicate that female presence stimulates GnIH release by the increase in NE release in the PVN, resulting in decreases in circulating LH and T levels in male quail (28) (Figure 2).

## Role of GniH in hypothyroidism-induced delayed puberty

Thyroid disorder is known to be associated with abnormal pubertal development. However, the mechanism of thyroid hormone (TH) action on pubertal onset remains unclear, although interactions between the hypothalamic-pituitary-thyroid (HPT) and HPG axes have been suggested (166–168) (Figure 3). Recently, Tsutsui and colleagues challenged this possibility by testing TH-mediated regulation of the HPG axis will be initiated by the change in the expression of GnIH, which acts at the most upstream level of the HPG axis by inhibiting the activity of GnRH neurons to reduce gonadotropin secretion from the pituitary gonadotropes [for reviews, see (15–18, 21–23, 44)] (Figure 3). To investigate the possible role of GnIH as a novel mediator between the HPT and HPG axes, Kiyohara et al. (169) investigated if abnormal thyroid status has an effect on pubertal onset and the HPG axis in female mice. Long-term treatment of the female mice with propylthiouracil (PTU) induced hypothyroidism and significant delay in pubertal onset (169). Importantly, hypothalamic GnIH mRNA expression is increased in hypothyroid female mice (169). Furthermore, circulating LH and estradiol-17β (E2) levels decreased in hypothyroid animals (169). It is therefore considered that pubertal onset may be delayed by hypothyroidism through the increase in GnIH expression and the decrease in LH and E2 levels in female mice (169) (Figure 3).

Kiyohara et al. (169) induced hypothyroidism in GnIH-knockout (KO) female mice, in order to demonstrate the involvement of GnIH in hypothyroidism induced pubertal disorder (169). Administration of PTU to GnIH-KO mice induces hypothyroidism with the reduced level of triiodothyronine, T_3_ (169). However, delayed puberty induced by hypothyroidism observed in wild type (WT) female mice was not observed in PTU-administered GnIH-KO female mice (169). Accordingly, it is considered that GnIH mediates hypothyroid induced delayed pubertal onset (Figure 3).

Molecular studies were further conducted to clarify the regulatory mechanism of GnIH on hypothyroidism-induced delayed puberty, as follows. Firstly, it was found that GnIH neurons in the hypothalamus express TH receptors (TRα and TRβ) (169) so that TH signals can be directly conveyed to GnIH neurons by TRs. Secondly, several putative TH-response elements (TREs) exist within 3 kb upstream from the mouse *GnIH* ORF. However, both TRs do not directly bind these TREs by chromatin immunoprecipitation (ChIP) assays (169), suggesting that TH (T_3_) may act *via* non-genomic action by membrane TRs. Importantly, H3 acetylation (H3Ac) correlated with gene activation is increased in hypothyroid female mice compared to control mice (169). Accordingly, thyroid status may modify chromatin structure of the *GnIH* promoter region resulting in the change in *GnIH* gene expression.

Some papers show that the elevated TRH level in hypothyroidism induces hyperprolactinemia and changes GnRH pulsatile secretion, leading to delayed LH response and delayed puberty (167). Other papers report that the increased TSH level activates gonadal function by the stimulation of receptor in gonads that is responsible for precocious puberty, as the structure of and receptors is similar (166, 168). However, the mechanism of how TH acts on the axis was not elucidated, although these papers suggest the presence of a mediator in the interaction of and HPG axes (166–168). Kiyohara et al. (169) found that thyroid dysfunction increases GnIH expression in the hypothalamus through chromatin modification of the promoter region of the *GnIH* gene in female mice. These results propose a novel function of GnIH serving as a mediator between the HPT and HPG axes (Figure 3). Female mice with hypothyroidism show pubertal delay with the increase in GnIH expression. Kiyohara et al. (169) further found that GnIH mediates the effect of hypothyroidism on pubertal delay, because delayed puberty was not observed in hypothyroidism induced GnIH-KO female mice. Accordingly, it is considered that GnIH is a critical factor that mediates abnormal thyroid status effect on pubertal onset [for a review, see (26)] (Figure 3).

## Conclusions

GnIH is a novel hypothalamic neuropeptide that inhibits gonadotropin synthesis and release. Studies on GnIH in the past 17 years have demonstrated that GnIH is highly conserved among vertebrates from agnathans to humans, which acts as a key neuropeptide inhibiting reproduction across vertebrates. GnIH inhibits gonadotropin synthesis and release through actions on gonadotropes and GnRH neurons via GPR147, the GnIH receptor. Thus, the discovery of GnIH has markedly advanced the progress of reproductive neuroendocrinology. Recent studies have further indicated that GnIH is involved in pubertal disorder induced by thyroid dysfunction. This is a novel function of GnIH mediating the interaction of the HPT-HPG axes in puberty.

Kisspeptin was discovered in mammals following the discovery of GnIH. GnIH and kisspeptin are new members of the RFamide peptide family, which act on the HPG axis to down-regulate and up-regulate the reproductive system, respectively. Therefore, we can now say that GnRH is not the sole hypothalamic neuropeptide that regulates reproduction. In the hypothalamus, GnIH neurons also projects to kisspeptin neurons. Importantly, GnRH and kisspeptin neurons both express GnIH receptor. Further studies may reveal unknown interactions among GnIH, GnRH and kisspeptin.

Furthermore, GnIH acts on the pituitary as well as the brain to regulate not only reproduction but also reproductive behaviors in vertebrates. GnIH activates P450arom and increases synthesis of neuroestrogen in the brain. GnIH may also change other neurosteroids' formation by activating or inactivating various steroidogenic enzymes. Steroidogenic enzymes are not only expressed in the brain but also expressed in the pineal gland, which is an endocrine organ located closely to the brain, and actively produces various neurosteroids *de novo* from cholesterol (170–174). As GnIH receptor is expressed in the pineal gland (Sato, M., Narihiro, M., Ubuka, T., Haraguchi., S., Tsutsui., K., unpublished observation), GnIH may regulate neurosteroidogenesis in the pineal gland as in the brain. Future studies are required to develop the emerging concept that the hypothalamic neuropeptide GnIH may modify neurosteroid synthesis in the brain and pineal gland to regulate brain functions.

## Author contributions

All authors listed have made a substantial, direct and intellectual contribution to the work, and approved it for publication.

### Conflict of interest statement

The authors declare that the research was conducted in the absence of any commercial or financial relationships that could be construed as a potential conflict of interest.
